# Complete genome sequence of *Pseudomonas aeruginosa* phage OmaKarin

**DOI:** 10.1128/mra.00353-26

**Published:** 2026-05-18

**Authors:** Jessica Ransome, Enea Maffei, Alexander Harms

**Affiliations:** 1 Institute of Food, Nutrition and Health, D-HEST, ETH Zurich 31089 , Zurich, Switzerland; Portland State University, Portland, Orlando, USA

**Keywords:** bacteriophage, *Casadabanvirus*, *Pseudomonas aeruginosa*, IS element

## Abstract

Bacteriophage OmaKarin was isolated on a restriction-deficient variant of the *Pseudomonas aeruginosa* PAO1 laboratory strain. We sequenced its 37,543 bp genome and classified it within the *Casadabanvirus* genus of temperate phages. Interestingly, a transposon has disrupted the lysogeny control module of OmaKarin, possibly explaining its apparent lack of temperate behavior.

## ANNOUNCEMENT

**Fig 1:**
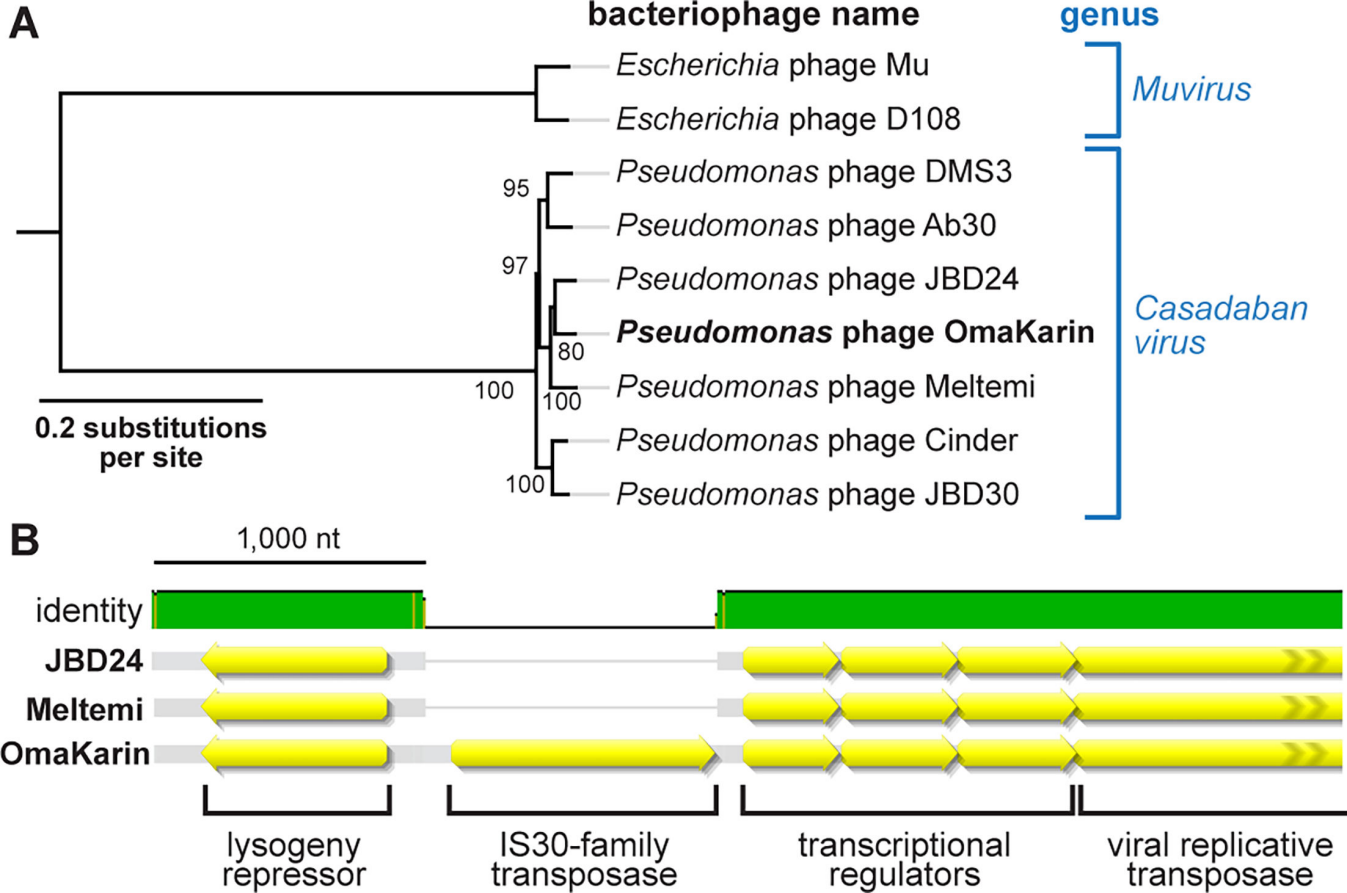
Whole-genome phylogeny of phage OmaKarin and disruption of its lysogeny control (**A**) The genomes of diverse *Casadabanvirus* phages were aligned using MAFFT (v7.490) implemented in Geneious Prime 2024.0.2 with FFT-NS-1 algorithm, 200PAM/*k* = 2 scoring matrix, gap open penalty 1.53, and offset value 0.123 (default settings; alignment length 22,175 nt) (16). A maximum likelihood phylogeny was calculated based on this alignment using PhyML (version 3.3.20180621) (17) implemented in Geneious Prime 2024.0.2 with the HKY85 substitution model and 100 bootstrap replicates. The phylogeny was rooted using *Muvirus* phages Mu and D108 as outgroup. Bootstrap values are shown if >70/100, and phage OmaKarin is highlighted in bold. (**B**) Comparison of the lysogeny control region of phage OmaKarin with the corresponding locus of two close relatives. The genomes were aligned using MAFFT (v7.490) implemented in Geneious Prime 2024.0.2 with the FFT-NS-1 algorithm, 200PAM/*k* = 2 scoring matrix, gap open penalty 1.53, and offset value 0.123 (default settings; displayed alignment length 3,200 nt) (16). Each genome is indicated as a gray bar with genes highlighted as yellow arrows. The sequence identity graph shows the identity between the three loci at each base position with a sliding window of 9, but the three sequences are almost identical (green: 100% identity, yellow: 30–99% identity, and red: <30% identity). Across the whole genome, the pairwise sequence identities of OmaKarin to JBD24 and Meltemi are 93.5% and 87.1%, respectively.

Temperate phages are viruses infecting bacteria that can integrate into the host genome as a prophage to establish a transiently mutualistic relationship known as lysogeny (1). A dedicated genetic module of the virus controls the formation and maintenance of lysogeny by silencing most prophage genes (1). Temperate phages of the genus *Casadabanvirus* are common viruses infecting *Pseudomonas aeruginosa* (2), including model phage DMS3 (3). Here, we report the genome of a *Casadabanvirus* called OmaKarin that has been isolated in 2023 from Swiss wastewater (sewage treatment plant ARA Canius in Lenzerheide, 46°42′33.3″ N, 9°33′05.7″ E). Briefly, wastewater had been concentrated with ZnCl_2_ before plating onto a lawn of host bacteria using a double-agar overlay with LB agar at 37°C as described previously (4). As isolation host, we used a mutant of the *P. aeruginosa* PAO1 laboratory strain in which we had previously inactivated its type I restriction-modification system as a possible obstacle for restriction-sensitive phages (5). One clear plaque was randomly selected during the filming for a documentary (6), and the phage isolate was passaged three times through single plaques for clonal purification. We then generated a high-titer lysate in SM buffer from a fully lysed double-layer agar lawn of the isolation host (7). For genome sequencing, viral DNA was extracted from 1 mL of high-titer stock using the Norgen Biotek Phage DNA isolation kit. Sequencing libraries were prepared with a streamlined tagmentation protocol using smaller volumes, cost-effective reagents, and fewer steps (8) before sequencing paired-end reads on a NovaSeq X Plus platform. After sequencing, we removed adapter sequences using bcl2fastq (v2.20.0.422; Illumina Inc., San Diego, USA) and then assembled 62,366 reads (151 bp length) using the Geneious Assembler implemented in Geneious Prime 2024.0.2 with additional trimming of terminal read regions showing >1% error probability per base. This resulted in one linear contig with 202× read coverage that we tentatively identified as the genome of a *Casadabanvirus* phage using BLAST (v.2.16.0) based on high sequence similarity to other viruses of this genus (9, 10). Due to their replication by transposition across the host chromosome (11), the consensus assembly of OmaKarin showed characteristic sharp edges around the true viral genome with highly divergent read sequences from the host chromosome on the distal sides. After removing these, the genome of OmaKarin has a length of 37’543 bp with 64.3% GC content, in which we could annotate 57 genes with Pharokka v1.7.3 using standard settings (12). Phylogenetic analyses confirmed the classification of OmaKarin as a *Casadabanvirus* (Fig. 1A). This was surprising since OmaKarin grows in clear plaques while temperate phages typically form turbid plaques (13). However, the lysogeny control module of OmaKarin is disrupted by a transposon, which may compromise stable prophage formation and maintenance (Fig. 1B). This transposon could be identified as an ISPpu17 element (IS30 family) using TnCentral (14), and virtually identical elements are common among pseudomonads including the first described ISPpu17 linked to antibiotic resistance of *P. putida* (14, 15). OmaKarin thus does not only expand the known diversity of *Casadabanvirus* phages but could also be an interesting model for the evolution of lysogeny.

## Data Availability

The genome sequencing project for OmaKarin has been deposited under accession number PRJEB110275 in the European Nucleotide Archive (ENA) database with the genome version described in this paper as the original (BioSample accession SAMEA121983792, Assembly accession GCA_982319625, and Reads accession ERR16878753).
